# Molecular and cellular mechanisms of chemoresistance in paediatric pre–B cell acute lymphoblastic leukaemia

**DOI:** 10.1007/s10555-024-10203-9

**Published:** 2024-08-05

**Authors:** Caleb B. Lill, Stephen Fitter, Andrew C. W. Zannettino, Kate Vandyke, Jacqueline E. Noll

**Affiliations:** 1 Myeloma Research Laboratory, School of Biomedicine, Faculty of Health and Medical Sciences, University of Adelaide, Adelaide, Australia; 2 Precision Cancer Medicine Theme, Solid Tumour Program, South Australian Health and Medical Research Institute, Adelaide, Australia

**Keywords:** Acute lymphoblastic leukaemia, Mesenchymal stromal cells, Adipocytes, Microenvironment, Chemoresistance

## Abstract

Paediatric patients with relapsed B cell acute lymphoblastic leukaemia (B-ALL) have poor prognosis, as relapse-causing clones are often refractory to common chemotherapeutics. While the molecular mechanisms leading to chemoresistance are varied, significant evidence suggests interactions between B-ALL blasts and cells within the bone marrow microenvironment modulate chemotherapy sensitivity. Importantly, bone marrow mesenchymal stem cells (BM-MSCs) and BM adipocytes are known to support B-ALL cells through multiple distinct molecular mechanisms. This review discusses the contribution of integrin-mediated B-ALL/BM-MSC signalling and asparagine supplementation in B-ALL chemoresistance. In addition, the role of adipocytes in sequestering anthracyclines and generating a BM niche favourable for B-ALL survival is explored. Furthermore, this review discusses the role of BM-MSCs and adipocytes in promoting a quiescent and chemoresistant B-ALL phenotype. Novel treatments which target these mechanisms are discussed herein, and are needed to improve dismal outcomes in patients with relapsed/refractory disease.

## Introduction

B cell precursor acute lymphoblastic leukaemia (B-ALL) is characterised by the rapid, uncontrolled proliferation of poorly differentiated B-lineage lymphoblasts within the bone marrow (BM), and is the most common paediatric malignancy, accounting for ~ 25% of all childhood cancer diagnoses [1]. An increased understanding of patient risk factors, through analysis of cytogenetics and genetics, allows patients to be separated into risk stratification groups, which subsequently dictates the intensity of therapy required for an optimal outcome [2]. This approach has increased the overall survival of B-ALL patients from 10–20% in the 1970s to 85–90% today [3]. However, despite highly effective current treatment strategies, 10–15% of patients will relapse, and more than 80% of this group will not respond to intensified chemotherapy, radiotherapy or allogeneic stem cell transplants and will ultimately die from the disease [4, 5]. Notably, novel treatments such as CD19- or CD22-targeted immunotherapy have significantly improved outcomes of high-risk or relapsed B-ALL [6–8]. However, despite these treatments, a number of paediatric B-ALL patients will still relapse. Resistance to chemotherapeutics is a hallmark of relapsed B-ALL [9–11]. A comprehensive understanding of the mechanisms underpinning chemoresistance will be critical in future attempts to treat relapse and improve outcomes for these patients.

Modern treatment regimens consist of three phases: (i) induction therapy, including chemotherapy to rapidly induce remission; (ii) consolidation therapy with more intense chemotherapy to target remaining B-ALL cells; and (iii) maintenance therapy to attempt to sustain remission. Specifically, a combination therapy consisting of vincristine, L-asparaginase (ASNase) and a glucocorticoid (dexamethasone or prednisolone), as well as an anthracycline (doxorubicin or daunorubicin), is generally used as the first-line treatment in B-ALL [12–15]. Intrathecal methotrexate is also included to reduce the likelihood of central nervous system relapse, which is the most common form of extramedullary relapse [16]. Depending on specific treatment protocols, additional agents used include cyclophosphamide, cytarabine and thiopurines [14, 15]. Notably, resistance to each of these therapeutics has been reported in B-ALL, with varying frequencies [9, 11, 17–21]. BM relapse is the most common form of relapse in B-ALL and is a consequence of the clonal expansion of B-ALL cells that remain after induction therapy [2]. However, it is unknown whether the presence of these residual tumour cells following therapy is caused by intrinsic chemotherapy resistance in subclones that have been present since diagnosis, or the acquisition of additional mutations that reduce chemotherapy sensitivity. Additionally, there is compelling evidence to suggest that protective niches within the BM microenvironment may play a key role in the protection of B-ALL cells from chemotherapy, leading to subsequent relapse. The BM is populated by a variety of cell types, including BM mesenchymal stromal cells (BM-MSCs), adipocytes, osteoblasts, osteoclasts, haematopoietic stem cells (HSC) and immune cells (among others) [22, 23]. Interactions between the cells of the BM microenvironment and B-ALL blasts are not only critical for leukaemogenesis [24], but also contribute to chemotherapy resistance of B-ALL blasts. In this review, we describe the key cellular and molecular mechanisms that protect B-ALL blasts from common chemotherapeutics and highlight some of the current research into therapies that may overcome chemoresistance.

## The role of BM-MSCs in B-ALL chemotherapy resistance

**Fig. 1:**
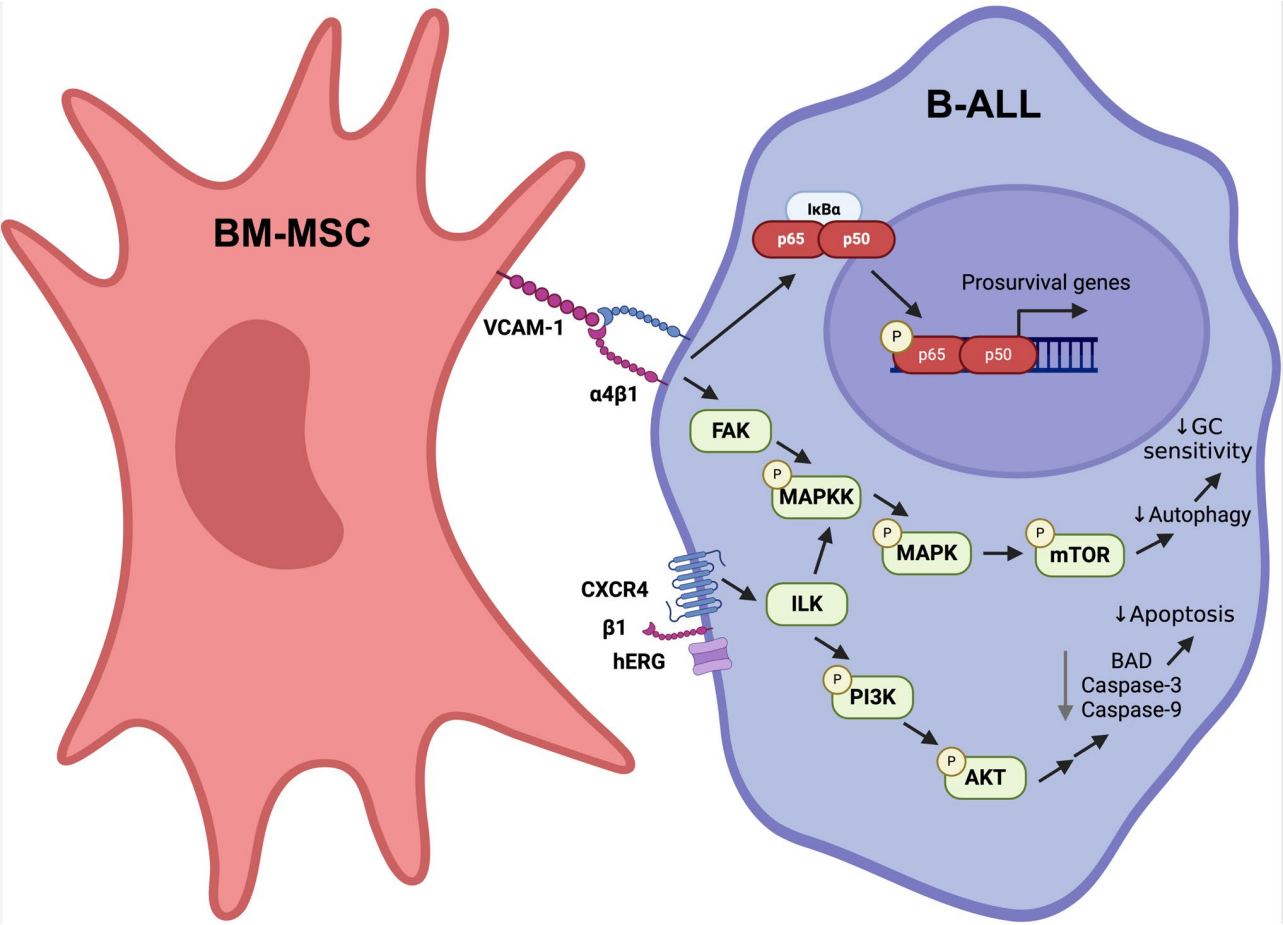
Integrin-mediated signalling promotes B-ALL chemoresistance. α4β1-VCAM-1 ligation induces expression of pro-survival signalling molecules *via* NF-κB activation and modulates mTOR activity *via* the MAPK/ERK signalling pathway to reduce B-ALL glucocorticoid sensitivity. Complexes composed of integrin β1, CXCR4 and hERG reduce activity of apoptosis-promoting proteins *via* ILK/PI3K/AKT signalling. VCAM-1, vascular cell adhesion molecule-1; FAK, focal adhesion kinase; MAPKK, mitogen-activated protein kinase kinase; MAPK, mitogen-activated protein kinase; mTOR, mechanistic target of rapamycin; CXCR4, CXC-motif chemokine receptor type 4; hERG, human ether-a-go-go-related gene; PI3K, phosphoinositide 3-kinase; BAD, beclin-2-associated death promoter protein; GC, glucocorticoid

BM-MSCs are multipotent progenitor cells which differentiate into cell types crucial for maintenance of the BM microenvironment, including osteoblasts, chondrocytes and adipocytes [25]. Extensive literature recognises that BM-MSCs support solid tumours that metastasise to the BM by increasing their growth and survival, as previously reviewed by Li et al. [26], and induce resistance to common chemotherapeutics [27]. Similarly, BM-MSCs also play a significant role in leukaemia, wherein their interactions with tumour cells within the BM have been shown to reduce the effectiveness of chemotherapy in both B-ALL [28] (summarised in Fig. 1) and AML [29].

### CXCL12/CXCR4 signalling

It is well documented that B-ALL cell migration is mediated, at least in part, by CXC-motif chemokine ligand 12 (CXCL12) binding to its receptor, CXC-motif chemokine receptor 4 (CXCR4) on B-ALL cells [30]. Specifically, the CXCL12/CXCR4 axis promotes migration to, and interactions with, BM-MSCs [31]. Of note, high CXCR4 expression and phosphorylation have been associated with poor overall survival in adult B-ALL [32, 33] and CXCR4 is upregulated in relapsed paediatric B-ALL and is predictive of extramedullary organ infiltration [34, 35].

Within the healthy BM microenvironment, BM-MSC secretion of CXCL12 and surface expression of vascular cell adhesion molecule-1 (VCAM-1) regulate the retention and quiescence of haematopoietic stem cells (HSCs) [36, 37]. Current evidence suggests that similar interactions take place between BM-MSC and B-ALL blasts, driving pro-survival signalling pathways and the acquisition of a quiescent phenotype [38], which, in turn, is likely to play a role in enhancing chemoresistance of B-ALL blasts (discussed below). Furthermore, the CXCR4/CXCL12 axis is known to increase integrin α4β1- and α5β1-mediated adhesion to VCAM-1, which also contributes to B-ALL chemoresistance (as discussed below) [39].

Inhibition of CXCR4 signalling with a specific inhibitor, AMD3100 (plerixafor), increases apoptosis in primary B-ALL cells treated with vincristine in a BM-MSC coculture system. Specifically, expression of anti-apoptotic Beclin-2 was decreased and pro-apoptotic Beclin-2-like-protein-4 was increased following AMD3100 treatment [34]. This compound is used in humans to mobilise HSCs to peripheral blood for collection prior to autologous stem cell transplant in non-Hodgkin’s lymphoma and multiple myeloma [40]. This may also be exploited for the treatment of chemoresistant B-ALL as studies have shown that AMD3100 treatment in a paediatric B-ALL xenograft model results in increased leukaemic mobilisation to the peripheral blood and enhanced effectiveness of vincristine treatment [41]. However, to date, while clinical trials for plerixafor have been performed/are underway in relapsed/refractory AML, this is yet to be investigated in paediatric B-ALL.

### Integrin-mediated cell–cell adhesion

Cellular adhesion within the BM is primarily mediated by integrins *via* the formation of heterodimers composed of α and β subunits [42]. Within the healthy BM microenvironment, HSCs express various integrin complexes including integrins α4β1, α5β1, α6β1 and α9β1, which interact with extracellular matrix (ECM) proteins such as osteopontin, laminin and fibronectin, as well as the cell surface protein VCAM-1, to regulate HSC homing and retention within the BM [43–47]. B-ALL blasts similarly express α4β1, α5β1 and α6β1, which facilitate adhesion to BM-MSC *via* VCAM-1, [48] as well as extracellular osteopontin [49], interactions that are reported to promote the survival and proliferation of B-ALL blasts [50]. Notably, Mudry et al. show that coculture with BM-MSCs *in vitro* reduces sensitivity of B-ALL cell lines to cytarabine and etoposide, while addition of VCAM-1-neutralising antibodies to the coculture system negates this protective effect [20]. Importantly, elevated mRNA expression of *ITGA4* (integrin α4) within patient BM aspirates is associated with poorer relapse-free and overall survival [51]. *ITGA4* mRNA expression is also significantly higher in patients who progress to relapse compared to those with continuous complete remission [51]. Furthermore, deletion of the α4 integrin subunit in murine B-ALL cells transformed with BCR-ABL reduced adhesion to recombinant murine VCAM-1, and increased sensitivity to vincristine/dexamethasone/ASNase treatment [52]. Similarly, treatment with a combination of an α4-neutralising antibody and vincristine/dexamethasone/ASNase in mice xenografted with chemotherapy-resistant primary B-ALL cells significantly increased overall survival relative to chemotherapy treatment alone. In addition, α4β1-neutralising antibodies in a human stroma/B-ALL cell line coculture model increased sensitivity of B-ALL cells to methotrexate and cytarabine [53]. Together, these results support a protective role for α4β1-mediated adhesion of B-ALL blasts to BM-MSC and/or ECM proteins, reducing their sensitivity to a variety of chemotherapeutic agents.

#### NF-κB signalling

Mechanistically, α4β1/VCAM-1 interactions promote NF-κB signalling [54]. Coculture of a human B-ALL cell line with primary BM-MSCs has been shown to significantly increase the localisation of the NF-κB p65 subunit to the nucleus of B-ALL cells [48]. Additionally, inhibition of NF-κB activation resulted in increased sensitivity to vincristine in B-ALL cells cocultured with BM-MSCs, suggesting that NF-κB activation, following B-ALL/BM-MSC interactions, contributes to chemotherapy resistance [48]. Furthermore, knockdown of p65 in REH and NALM6 B-ALL cell lines enhanced the *in vivo* sensitivity of B-ALL cells to cytarabine treatment in xenograft models [55]. Integrins αvβ3 and αvβ5 on B-ALL cells similarly mediate adhesion to BM-MSC-expressed ECM protein, periostin [56]. This interaction promoted NF-κB activation in a B-ALL cell line, consequently increasing B-ALL proliferation and CCL2 production, which further increased BM-MSC periostin expression [56]. Broadly, these results provide evidence that induction of NF-κB signalling, mediated by α4β1/VCAM-1, αvβ3/periostin and αvβ5/periostin interactions in B-ALL cells, may contribute to reducing the cytotoxic effects of commonly used B-ALL chemotherapeutics.

#### PI3K/AKT activation *via* hERG/β1 integrin signalling

The formation of a membrane-bound complex containing the β1 integrin, CXCR4 and the human ether-a-go-go-related gene (hERG) potassium channel, on the surface of B-ALL cells following B-ALL/BM-MSC interactions, contributes to chemoresistance [57]. This complex promotes activation of the integrin-linked kinase (ILK), which is critical for BM-MSC-induced pro-survival PI3K/AKT signalling [50]. Indeed, E4031, a hERG1 inhibitor, promoted B-ALL cell death and worked synergistically with doxorubicin, methotrexate and prednisolone treatments in cocultures of primary B-ALL cells or cell lines with BM-MSCs [57]. Notably, inhibition of β1 integrin or hERG1 activity reduced AKT phosphorylation and E4301 abrogated the protective effect of BM-MSC coculture on B-ALL chemosensitivity, providing further support for the contribution of the hERG1/β1 integrin complex in adhesion-mediated chemoresistance. Mechanistically, activation of the PI3K/AKT pathway suppresses activation of apoptotic pathways in B-ALL [58]. Specifically, activated AKT phosphorylates and inactivates the pro-apoptotic protein BAD (Beclin-2-associated death promoter) [59], and inhibits activation of the downstream apoptosis-regulating proteins caspase-3 and caspase-9 [60]. Activation of AKT has been associated with poor prognosis and chemotherapy resistance in B-ALL, and AKT overexpression in the human B-ALL cell line NALM6 reduces sensitivity to numerous chemotherapeutic agents *in vitro* [61]. Together, these results provide evidence for the role of integrin-mediated signalling *via* the PI3K/AKT pathway in promoting B-ALL cell survival during chemotherapy treatment (summarised in Fig. 1).

Molecules which target the CXCR4/β1 integrin/hERG1 complex may be attractive options for treatment of chemoresistant and/or relapsed B-ALL. However, targeting of potassium ion channels, such as hERG1, is challenging due to the risk of significant side effects, as inhibition disrupts the heart sinus rhythm leading to arrythmia and cardiotoxicity [62]. Hence, first-generation hERG1 inhibitors such as clofilium and cisapride are unlikely candidates for treatment of relapsed/chemoresistant B-ALL. Notably, a more selective hERG1 inhibitor, CD-160130, blocks hERG1 function without inducing detectable cardiotoxicity *in vivo* [63]. Crucially, CD-160130 enhanced doxorubicin cytotoxicity in B-ALL cell lines and patient samples, and abolished the chemoprotective effect of BM-MSC coculture [63]. Hence, this molecule could be investigated as a potential treatment for relapsed B-ALL.

#### MAPK/ERK signalling

Signalling *via* integrin β1 complexes results in the activation of downstream signalling cascades involving ILK [57] and focal adhesion kinase (FAK) [64, 65], which in turn lead to activation of the MAPK/ERK signalling pathway. Indeed, activation of MAPK/ERK has been observed in both B-ALL and BM-MSC coculture studies *in vitro* [66] and in *ex vivo* cultured patient samples following stimulation with CXCL12 [67].

Current evidence suggests glucocorticoids induce cell death of B-ALL blasts by first triggering autophagy, which in turn promotes apoptosis [68, 69]. Hence, signalling pathways which prevent autophagy in B-ALL blasts may also contribute to glucocorticoid resistance. Indeed, MAPK/ERK signalling has been identified as a key factor which modulates mTOR activity and inhibits autophagy induction, thereby reducing leukaemic cell death [70]. Notably, elevated MAPK/ERK pathway activation, as reflected by increased phosphorylated ERK levels by flow cytometry, has been identified in relapsed patient samples when compared with samples from newly diagnosed patients. Furthermore, knockdown of the MAPK/ERK pathway activator, mitogen-activated protein kinase kinase (MEK), in glucocorticoid-resistant B-ALL cell lines restored drug sensitivity [71]. In addition, primary B-ALL blasts from relapsed patients exhibited increased cell death relative to matched diagnostic samples when treated with a MEK inhibitor, which in turn worked synergistically with glucocorticoid treatment in relapse samples [71]. These findings suggest that MAPK/ERK signalling is associated with relapse in B-ALL and may play a role in glucocorticoid resistance.

Together, these findings support the use of molecules which target MAPK/ERK signalling in the treatment of glucocorticoid-resistant relapsed B-ALL. The MEK inhibitor, trametinib, is approved for human use and has been used successfully for clinical treatment of metastatic melanoma [72] and high-grade paediatric glioma [73]. While the previously discussed studies provide preliminary evidence for MEK inhibitor activity against glucocorticoid-resistant relapsed B-ALL, further studies are needed to assess the effectiveness of MEK inhibition in B-ALL patients. In addition, targeting alternative upstream regulators of the MAPK/ERK pathway may also improve the effectiveness of standard of care chemotherapeutics. Notably, ILK inhibition has been demonstrated to suppress both PI3K/AKT signalling and MAPK/ERK activation in prostate cancer and chronic lymphocytic leukaemia (CLL) cell lines [74–76]. Furthermore, an ILK inhibitor synergises with doxorubicin to induce apoptosis of bladder cancer cells [77]. Similarly, FAK inhibition synergises with dasatinib *in vitro* when used to treat Ph^+^ B-ALL [78]. There is significant evidence from *in vitro* and *in vivo* studies, as well as from analysis of patient samples, that pro-survival pathways downstream of integrin-mediated adhesion are likely to play a critical role in chemoresistance. Therefore, further investigation of inhibitors targeting these pathways is warranted.

### Tunnelling nanotube-mediated B-ALL chemoresistance

In addition to integrin-mediated signalling in B-ALL, the formation of tunnelling nanotubes (TNTs) between B-ALL cells and BM-MSCs may promote B-ALL chemoresistance [79]. TNTs are thin membrane protrusions stabilised by F-actin, which facilitate intercellular communication, and transfer of organelles and signalling molecules [80, 81]. These structures promote resistance in solid cancers [82, 83], AML [84] and T-ALL [85]. Notably, in a B-ALL coculture model, transfer of material from primary B-ALL cells to BM-MSCs, and vice versa, was observed [79]. In this study, inhibition of nanotube formation significantly reduced BM-MSC-mediated resistance to prednisolone in B-ALL. Furthermore, nanotube-dependent signalling from B-ALL cells to BM-MSC increased expression of chemoattractant molecules IL8, CCL2 and CXCL10 [79], which are known to promote migration towards, and adhesion to, BM-MSCs [86, 87], contributing to chemoresistance as discussed above. A subsequent study suggests that TNT-mediated transfer of mitochondria and autophagosomes from B-ALL cells promotes autophagy induction in BM-MSCs, thereby upregulating expression of the aforementioned cytokines [88]. Furthermore, BM-MSC transfer of mitochondria to B-ALL cells *via* TNTs reduced levels of reactive oxygen species (ROS) in B-ALL cells *in vitro*, and attenuated ROS-induced cell death following cytarabine treatment [89]. These findings suggest co-administration of nanotube-inhibiting agents (e.g. vincristine) with ROS-inducing chemotherapeutics (anthracyclines and cytarabine) may reduce nanotube-induced B-ALL chemoresistance.

### BM-MSC-induced B-ALL quiescence

As chemotherapies used to treat B-ALL primarily target proliferating, metabolically active cells, this suggests a hypothesis whereby the adoption of a quiescent phenotype by B-ALL blasts (a state in which blasts remain inactive and cease proliferation) could reduce sensitivity to cytotoxic agents [90]. This could then allow latent B-ALL blasts to persist after chemotherapy and subsequently cause relapse. Indeed, in patient-derived xenograft (PDX) models, rare populations of quiescent cells have been identified following chemotherapy that display characteristics of long-term dormancy, treatment resistance and stemness [91, 92]. Notably, these quiescent B-ALL cells displayed similar transcriptional characteristics to relapsed B-ALL [92], suggesting they are the source of relapse following initial chemotherapy.

Interactions within the bone microenvironment are critical for regulation of tumour cell quiescence as dormant, therapy-resistant blasts were shown to begin proliferating and become sensitive to treatment following dissociation from their *in vivo* environment [91]. Indeed, the specific localisation of B-ALL blasts within the bone is likely to be important. In a PDX model of B-ALL, non-cycling, quiescent B-ALL cells were found to localise in close proximity to endosteal surfaces, suggesting that these niches may induce quiescence in B-ALL cells [49]. Similarly, in a “leukaemia-on-a-chip” model, B-ALL cells in proximity to BM-MSCs displayed decreased proliferation, and those directly adhered to BM-MSCs did not express the proliferation marker Ki67 [38]. This induction of quiescence is suggested to be related to the upregulation of osteopontin (OPN) within BM-MSCs following interaction with B-ALL cells. Indeed, Boyerinas et al. showed co-localisation of quiescent B-ALL cells with OPN-expressing cells *in vivo* [49]. In this model, treatment with OPN-neutralising antibodies abrogated B-ALL/BM-MSC adhesion and increased tumour cell Ki67 expression and BM tumour burden, supporting the conclusion that OPN contributes to the quiescence of B-ALL blasts within the BM. Furthermore, OPN-neutralising antibodies worked synergistically with cytarabine treatment in mice, suggesting that the release from dormancy is sufficient to enhance chemosensitivity. Together these studies suggest that treatment outcomes may be improved by incorporating therapeutics which prevent adhesion to BM-MSC and the subsequent induction of quiescence within the endosteal niche.

### L-asparaginase inhibition

L-asparaginase (ASNase), which hydrolyses asparagine to aspartic acid and ammonia, is a key component of current standard therapeutic regimens for B-ALL. Notably, B-ALL cells are extremely sensitive to asparagine depletion by ASNase [93, 94]. Due to epigenetic silencing of the asparagine synthetase gene, *ASNS*, which encodes the enzyme responsible for converting glutamine and aspartate to asparagine, B-ALL cells themselves produce very little asparagine and as such are reliant upon external sources for their growth and survival [95, 96]. In addition, *ex vivo* sensitivity of patient leukaemic blasts to ASNase is strongly correlated with day 15 and day 42 minimal residual disease (MRD) [97], which in turn is a strong predictor of event-free survival [98]. Therefore, the sensitivity of B-ALL blasts to ASNase is likely an important contributor to individual patient therapeutic responses.

**Fig. 2:**
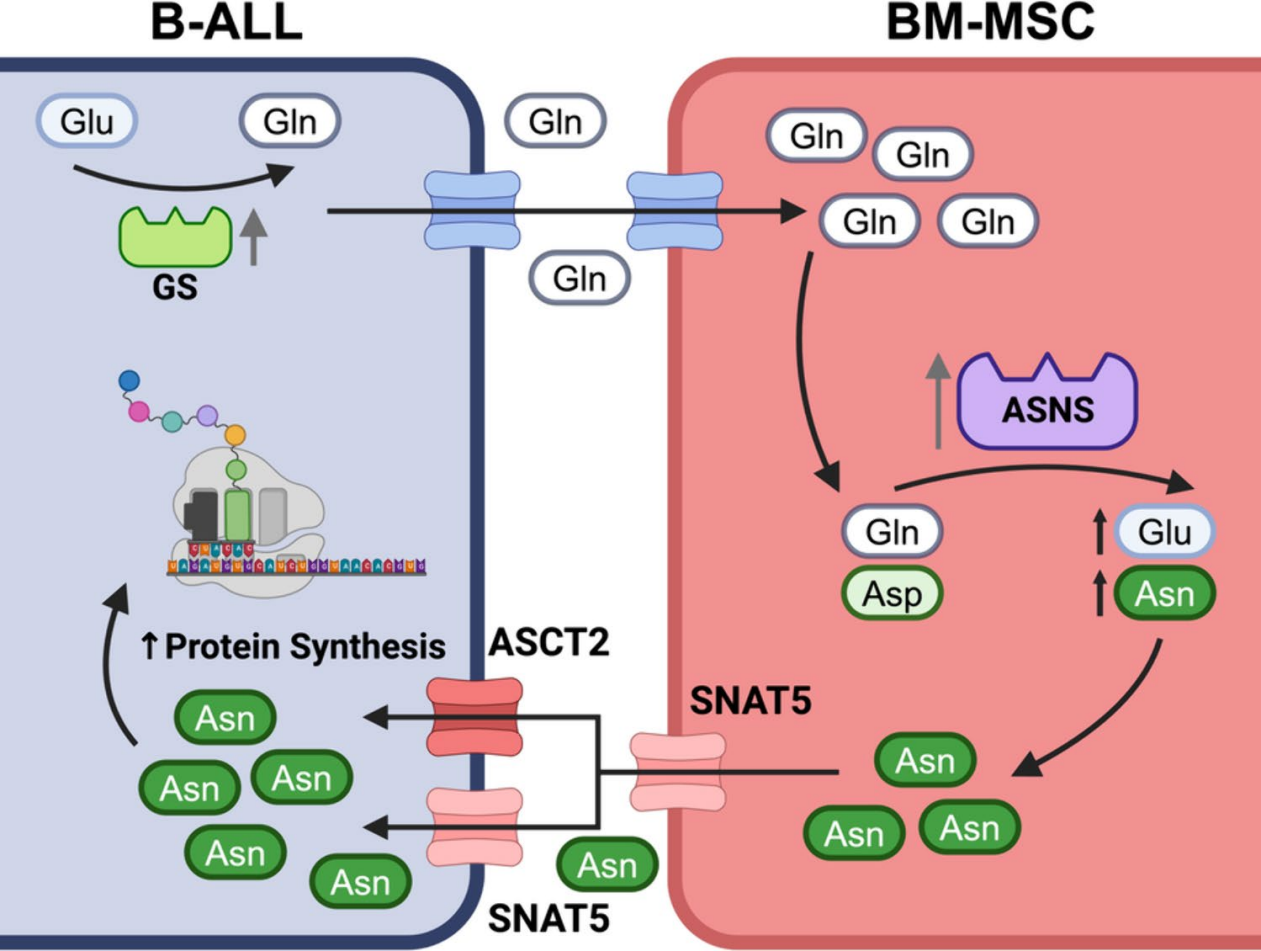
BM-MSCs supplement asparagine to overcome ASNase treatment cytotoxicity. Interactions with BM-MSC promote glutamine production and secretion by B-ALL blasts, which is subsequently converted to asparagine by ASNS and exported from BM-MSC *via* SNAT5, thereby reducing ASNase treatment effectiveness. SNAT5, sodium-coupled neutral amino acid transporter 5; ASCT2, alanine serine cysteine transporter 2; ASNS, asparagine synthetase; Asn, asparagine; Glu, glutamate; Gln, glutamine; Asp, aspartate; GS, glutamine synthetase

BM-MSCs have also been shown to modulate ASNase resistance. BM-MSCs are thought to be a key source of asparagine for B-ALL cells in the BM, as BM-MSCs display ~ 20-fold higher asparagine synthetase mRNA expression than primary B-ALL cells [99]. Interactions between B-ALL blasts and BM-MSC significantly decrease the cytotoxicity of ASNase in both human B-ALL cell lines and primary B-ALL cells, a function that was demonstrated to be dependent on BM-MSC *ASNS* expression and asparagine secretion [99, 100]. A recent study by Chiu et al. describes a BM-MSC/B-ALL interaction which reduces B-ALL chemosensitivity to ASNase by altering the glutamine/asparagine flux within these cells [100] (Fig. 2). Treatment of a B-ALL/BM-MSC coculture system with an irreversible glutamine synthase inhibitor, methionine-L-sulfoxamine (MSO), in combination with ASNase significantly reduced the viability of B-ALL cells when compared with ASNase treatment alone. It was also observed that ASNase treatment upregulated B-ALL expression of *GLUL*, the gene encoding glutamine synthase, and promoted B-ALL glutamine secretion. BM-MSCs were shown to take up and convert extracellular glutamine to asparagine, and subsequently secrete this asparagine *via* the amino acid transporter, SNAT5 [100]. Using the RS4;11 B-ALL cell line, a recent study identified SNAT5 and another neutral amino acid transporter, ASCT2, as essential for B-ALL cell uptake of asparagine from the extracellular environment [101]. Notably, inhibition of SNAT5 and ASCT2 in RS4;11 cells by glutamate-γ-monohydroxamate and V-9302, respectively, decreased intracellular asparagine concentrations and halted cellular proliferation. Furthermore, SNAT5 and ASCT2 inhibition in primary B-ALL cells resulted in greater cell death than ASNase treatment, despite no observed cytotoxicity to BM-MSCs at the same concentration [101]. Hence, these results suggest the existence of a pathway by which B-ALL blasts upregulate production and secretion of glutamine such that BM-MSCs can compensate for asparagine deficiency after ASNase treatment, thereby reducing B-ALL ASNase sensitivity (summarised in Fig. 2). Notably, a similar observation has been made in other malignancies, with glutamine synthase inhibition by MSO increasing the cytotoxicity of ASNase in multiple pancreatic cancer cell lines [102]. These findings support future research into the development of targeted therapeutics to treat ASNase resistance in B-ALL. Importantly, tolerance to MSO has been examined in multiple non-cancer mouse models without any overt toxicity [103–105]. In addition, an inhibitor for SNAT5, glutamate-γ-monohydroxamate, has been shown to reduce tumour burden in syngeneic mouse models of leukaemia and melanoma [106], suggesting that targeting of this pathway has therapeutic potential.

## The role of adipocytes in B-ALL chemotherapy resistance

**Fig. 3:**
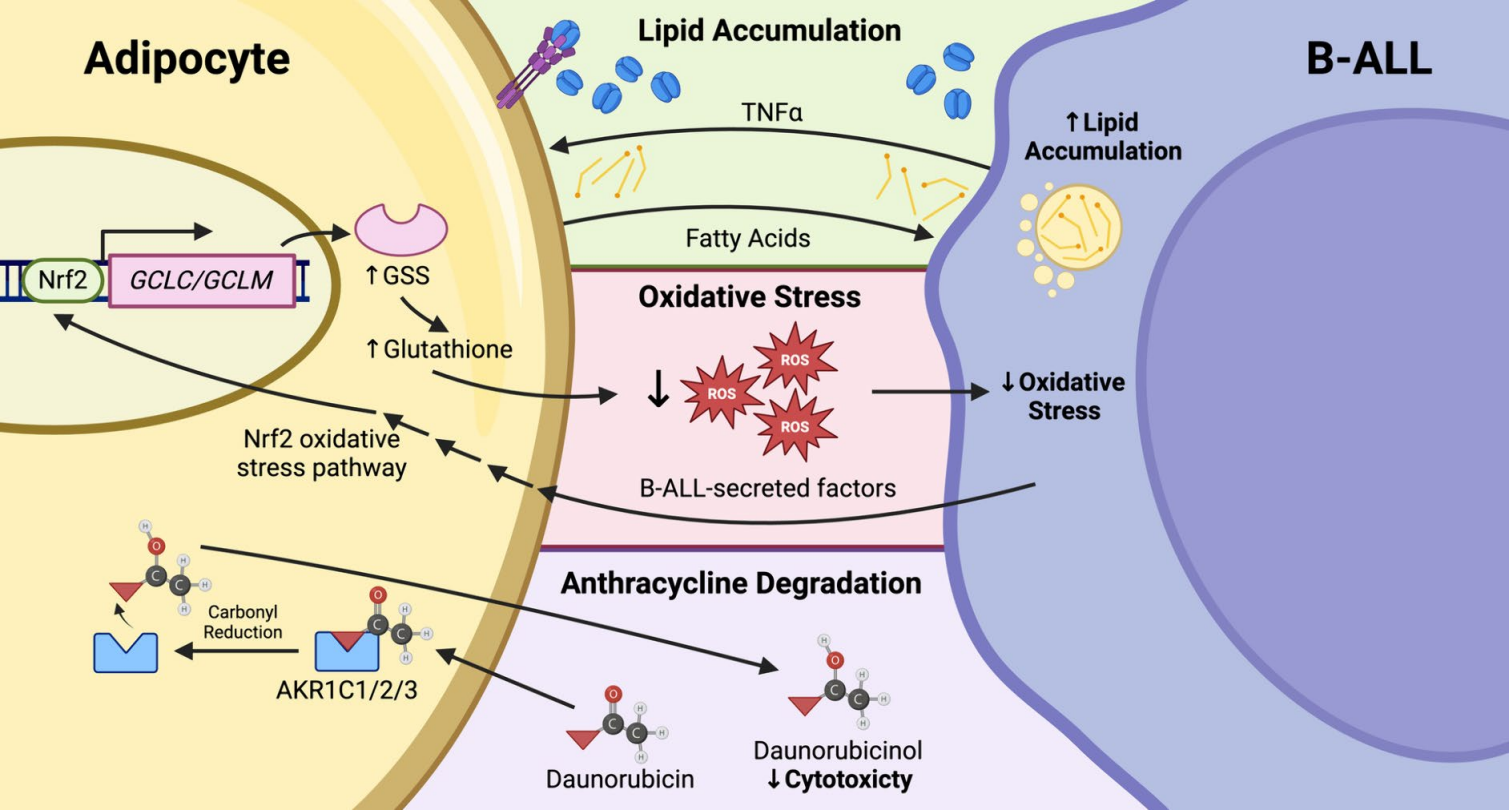
Adipocyte/B-ALL interactions contribute to B-ALL chemoresistance. B-ALL-secreted TNFα promotes secretion of lipids by adipocytes, which are used in fatty acid oxidation and decrease sensitivity to chemotherapeutics. Induction of the Nrf2-mediated oxidative stress pathway increases expression of the ROS scavenger, glutathione, thereby attenuating the effectiveness of therapeutics whose activity relies upon generating ROS (e.g. anthracyclines). Aldo-kedo reductases similarly reduce B-ALL anthracycline resistance by catalysing the formation of less cytotoxic derivatives such as daunorubicinol. ROS, reactive oxygen species; Nrf2, nuclear factor erythroid 2-related factor 2; GSS, glutathione synthetase; GCL, glutathione cysteine ligase; AKR, aldo–keto reductase; TNFα, tumour necrosis factor α

Adipocytes are a major cell type within the BM, making up approximately 50% of the total BM volume [107]. At homeostasis, the BM adipose tissue functions as an endocrine organ by secreting adipokines such as adiponectin and leptin that have systemic effects on metabolism [108, 109]. However, there is evidence to suggest that the release of adipokines and cytokines by adipocytes within the BM may also support tumour development in haematological cancers as well as bone metastasis of solid tumours [110]. Notably, BM-MSCs from B-ALL patients display an increased capacity to differentiate into adipocytes compared with normal donors [111], and adipocytes have been demonstrated to promote chemotherapy resistance in both solid and haematological cancers through multiple distinct mechanisms [111–119] (summarised in Fig. 3).

In B-ALL, obesity correlates with detectable MRD following treatment [120], a known prognostic marker for B-ALL outcomes [98, 121], and is associated with a poorer overall and relapse-free survival [122, 123]. The relationship between adipocytes and chemoresistance in B-ALL was first reported by Behan et al., who established a syngeneic diet-induced obesity model within C57BL/6 mice injected with the murine Ph^+^ B-ALL cell line 8093 [117]. These early studies demonstrated that obese mice had significantly poorer overall survival relative to controls following vincristine treatment.

Subsequent *in vitro* apoptosis assays demonstrated that 8093 cells cocultured with a murine preadipocyte cell line, 3T3-L1, exhibited enhanced resistance to vincristine treatment, an effect that was further increased following coculture with differentiated 3T3-L1 adipocytes. Similar results were observed for other common B-ALL therapeutics nilotinib, daunorubicin and dexamethasone [117]. A more recent study also showed resistance to anthracyclines in human B-ALL cell lines cocultured with a human adipocyte cell line [124]. Furthermore, it was shown that adipocyte-conditioned media alone was not sufficient to protect B-ALL cells from anthracyclines, while conditioned media from an adipocyte/B-ALL coculture demonstrated a protective effect [115]. This suggests that the direct cell–cell interaction between B-ALL cells and adipocytes is required for the secretion of factors which in turn contribute to the observed adipocyte-mediated chemoprotective effect.

### Free fatty acids

There is evidence to suggest that B-ALL cells produce factors that reprogram BM adipocytes to release free fatty acids (FFA), which in turn provides a favourable BM niche for leukaemic cell survival and likely serves as an additional source of energy [111, 125]. A recent study found that B-ALL conditioned media stimulated the release of FFA from adipocyte-differentiated 3T3-L1 cells [125]. When recombinant tumour necrosis factor α (TNFα) was used to simulate B-ALL TNFα secretion, a similar effect was observed, which was reduced by treatment with a TNFα inhibitor [125]. This suggests B-ALL cells may induce the release of FFA from adipocytes *via* TNFα secretion. Furthermore, supplementing B-ALL cells with oleic acid, the second most abundant FFA within the BM microenvironment, modestly reduced the cytotoxicity of low-dose vincristine and daunorubicin treatments *in vitro* [125–127]. In addition, it has been reported that ASNase treatment inhibits mTORC1 signalling in B-ALL cells, thereby reducing glucose metabolism and increasing reliance on FFA oxidation [128]. Intriguingly, inhibition of FFA oxidation in B-ALL cell lines significantly increased sensitivity to ASNase treatment. Similar results have been observed in other leukaemias, where inhibition of FFA oxidation increased the sensitivity of T-ALL [129] and chronic lymphocytic leukaemia [130] cells to glucocorticoids. Together, these findings suggest that leukaemic cells may exploit sources of exogenous lipids, including those secreted by adipocytes, to attenuate cytotoxicity of chemotherapeutics.

### Glutamine metabolism

The current ASNases approved for clinical use also display some glutaminase activity, albeit much lower than their asparaginase activities [131]. Despite this, recent findings provide evidence that the glutaminase activity of ASNases is important for the cytotoxic effects on B-ALL cells, as mutant ASNases with abolished glutaminase activity were significantly less cytotoxic to B cell lymphomas when compared to WT ASNases [132, 133]. Indeed, adipocytes are a major source of glutamine within the BM, and coculture of B-ALL cells and adipocyte-differentiated 3T3-L1 cells results in a two-fold increase in viability and proliferation relative to those cocultured with undifferentiated 3T3-L1 fibroblasts [116, 118]. This effect was abrogated by pre-treatment of adipocytes with a glutamine synthetase inhibitor, providing further evidence that adipocyte-derived glutamine reduces the effectiveness of ASNase treatment [116]. The mechanism underlying this effect is unknown, however it is plausible that adipocyte ASNS converts glutamine to asparagine to support B-ALL cells during ASNase treatment. Some opinions suggest residual ASNS activity within some B-ALL blasts is sufficient to reduce ASNase toxicity in the presence of glutamine [134]. Nevertheless, targeting glutamine synthesis to reduce ASNase resistance is an attractive therapeutic strategy. Unfortunately, to date, targeting glutamine metabolism of B-ALL patients with L-glutamine analogues has proved ineffective, and is frequently associated with neurotoxicity and hepatotoxicity in patients [135].

### Adipocyte-induced B-ALL quiescence

Similarly to BM-MSCs, adipocytes have also been shown to induce a quiescent phenotype in B-ALL blasts [136]. Coculture of human B-ALL cell lines with primary human BM adipocytes resulted in arrest of B-ALL cells in the G_0_ cell cycle phase [111]. Furthermore, in PDX models, B-ALL cells within the adipocyte-rich tail BM or gonadal adipose were significantly more quiescent relative to those within the relatively adipocyte-poor femur BM. This effect was consistent across four genetically distinct PDX and was speculated to be caused by adipocyte-induced suppression of protein synthesis [111]. In addition, a recent study identified that increased expression of galectin-9 on the cell surface of B-ALL blasts in response to adipocyte-secreted factors may induce cell cycle arrest in response to chemotherapy, potentially by modulating activity of cell cycle checkpoint regulators [137]. While these results establish a relationship between B-ALL quiescence and adipocyte interaction, further work is required to understand how adipocytes specifically induce quiescence of B-ALL blasts, and whether these mechanism(s) can be targeted to enhance the effectiveness of current treatment protocols.

### Adipocyte uptake and metabolism of anthracyclines

Adipocytes may also sequester anthracyclines, thereby protecting B-ALL cells by limiting their exposure. In coculture with murine adipocytes, human B-ALL cell lines treated with daunorubicin accumulated significantly lower cytoplasmic amounts of drug than those in monoculture, resulting in decreased B-ALL cell death [124]. Notably, a concomitant, dose-dependent increase of daunorubicin within the cytoplasm of adipocytes was observed, suggesting adipocytes sequester significant concentrations of daunorubicin from the environment. A similar phenomenon has been noted in other cancers, with uptake of a closely related anthracycline, doxorubicin, also observed in human adipocytes differentiated from BM-MSCs, resulting in protection of a multiple myeloma cell line from cytotoxicity [138]. Similarly, adipocytes were shown to promote doxorubicin resistance of breast cancer cell lines *in vitro via* increased drug efflux. Intriguingly, murine adipocytes cocultured with a human B-ALL cell line were shown to metabolise daunorubicin to its less toxic metabolite, daunorubicinol [124]. The enzymes responsible for this conversion, aldo–keto reductases and carbonyl reductases, are highly expressed in adipose tissue samples [139–142]. In addition, adipocyte-mediated degradation of daunorubicin was confirmed *in vivo* by analysing the daunorubicinol:daunorubicin ratio within adipose tissue from C57BL/6 mice after daunorubicin treatment [124]. While studies investigating this mechanism of chemoprotection are currently limited, these results suggest that metabolism of anthracyclines by adipocytes may protect B-ALL cells from their cytotoxic effects. However, further studies are required to confirm the relevance of this in B-ALL.

### Oxidative stress

Further evidence suggests that adipocytes support the survival of B-ALL cells by reducing the cytotoxicity of anthracyclines. Under standard conditions, anthracycline treatments promote the formation of ROS within B-ALL cells, which induce double-stranded DNA breaks and consequently lead to cell death [143–145]. Interestingly, when mouse or human B-ALL cells were cocultured with adipocytes and treated with daunorubicin, cell viability was significantly improved relative to monoculture [115]. This effect was hypothesised to be due to induction of the oxidative stress pathway within adipocytes by B-ALL-secreted factors [115]. Specifically, adipocyte expressions of glutathione cysteine ligase subunits (*GCLC* and *GCLM*), which are responsible for catalysing the formation of the ROS-scavenging glutathione (GSH), were significantly increased during coculture with B-ALL cells. Notably, elevated levels of GSH have been associated with poorer overall and event-free survival in B-ALL [146, 147]. Furthermore, genes involved in the Nrf2-mediated oxidative stress response pathway were upregulated within adipocytes in a B-ALL coculture system [115]. A similar mechanism has been observed in other haematological malignancies, with GSH attenuating ROS-mediated cell death following chemotherapy in AML [148], CML [149–151] and multiple myeloma [152, 153]. Therefore, it is suggested that increased adipocyte GSH synthesis may, at least in part, protect B-ALL cells from daunorubicin by reducing induction of oxidative stress pathways. However, further studies are required to verify the significance of increased adipocyte GSH synthesis in reducing oxidative stress in B-ALL blasts.

## Conclusion

Patient outcomes in paediatric B-ALL have improved significantly due to modern chemotherapeutic regimens. However, approximately 15% of patients will relapse due to the outgrowth of latent clones which treatment failed to eradicate. These patients have extremely poor prognosis, as clones that survive treatment are generally resistant to chemotherapeutics. While genetic factors are likely to play a role in chemoresistance (as reviewed by Jędraszek et al. [154]), it is evident from a large body of literature that chemoresistance is also driven by interactions between B-ALL blasts and accessory cells within the BM microenvironment, particularly BM-MSCs and adipocytes. Other cell populations within the BM microenvironment, such as immune cells, are also likely to play a role in chemoresistance. However, these populations are not discussed herein due to insufficient experimental data. Notably, adipocytes and BM-MSCs have been shown to modulate sensitivity to the key induction phase therapeutic, ASNase, *via* distinct mechanisms. Unsurprisingly, a number of these cell–cell interactions lead to the induction of pro-survival pathways that are commonly associated with therapy resistance in many solid and haematological cancers, including the CXCR4, PI3K/AKT, MAPK/ERK and NF-κB signalling pathways. Together, these pathways reduce cellular stress in response to chemotherapy and suppress the activation of pro-apoptotic pathways in B-ALL blasts. Interactions with both BM-MSCs and adipocytes have also been demonstrated to induce B-ALL cell quiescence, which may also result in reduced sensitivity to chemotherapeutics. Therefore, molecules that target interactions between B-ALL blasts and cells of the BM microenvironment represent attractive therapeutic options for preventing and/or treating relapse. As discussed above, there are numerous therapeutics that inhibit critical interactions and signalling pathways known to promote B-ALL chemoresistance; however, many are yet to be tested in the context of B-ALL. Of note, therapeutics which target the α4 or β1 integrins have shown promise both *in vitro* and in mouse xenograft models. Hence, further investigation of these therapeutics is warranted. Furthermore, molecules which attenuate integrin-mediated pro-survival signalling pathways following B-ALL/BM-MSC interactions, such as the PI3K/AKT and MAPK/ERK pathways, should also be examined. Targeting B-ALL/adipocyte interactions will likely improve overall survival of B-ALL patients. To achieve this, a more complete understanding of the mechanisms of adipocyte-induced chemoresistance in B-ALL is required.

## Data Availability

No datasets were generated or analysed during the current study.
